# An Internet Hospital Plus Home Nursing Model for Chronic Disease Patients: Mixed-Methods Study in Tianjin, China

**DOI:** 10.2196/76761

**Published:** 2025-11-05

**Authors:** Yuzhe Wang, Yingchun Liu, Ziqi Jin, Chunjie Jin, Fang Hu, Tianzhi Yu

**Affiliations:** 1 School of Management, Tianjin University of Traditional Chinese Medicine, Tianjin, China; 2 Healthcare Security Department, Tianjin Medical University Chu Hsien-I Memorial Hospital, Tianjin, China; 3 Information Department, Internet Hospital, Tianjin Medical University General Hospital, No. 154 Anshan Rd, Heping District, Tianjin, 300052, China, 86 022-60362218, 86 022-60361370

**Keywords:** internet hospitals, home nursing, services, effectiveness, mixed-methods

## Abstract

**Background:**

Internet hospitals and “Internet + nursing services” represent emerging medical and nursing models in China. These platforms integrate online systems with offline care to extend services beyond traditional hospital settings. With the rapid expansion of internet hospitals, a new model—internet hospital plus home nursing—has developed. However, research on its implementation and effectiveness remains limited.

**Objective:**

This study evaluates the implementation of the internet hospital plus home nursing model by analyzing workload, patient satisfaction, and nurses’ perceptions, aiming to provide a strategic reference for its further development.

**Methods:**

Data from 2459 patients who used internet hospital plus home nursing services were collected from a hospital database. The frequency of applications and service timeliness were analyzed using *χ*^2^ tests. Patient diagnoses, service types, and geographic distribution were summarized using frequency tables and visualization techniques. A simulation approach, along with the Mann-Whitney *U* test, was used to compare the costs of transferring patients to hospitals versus providing home nursing. Multiple linear regression identified factors associated with cost differences. Patient satisfaction across different stages was compared using a *U* test, and nurses’ attitudes were assessed via a questionnaire.

**Results:**

The majority of patients were aged 60 years and older (2120/2459, 86.2%). A significant difference in application frequency was observed across age groups (*χ*²_4_=29.86; *P*<.001). Oncology patients were the most common users (1468/7415, 19.8%) and intravenous blood collection was the most frequent service (4899/7415, 66.1%). Most patients resided within 6 regions near the physical hospital (2119/2459, 86.2%). All patients received services within 2 days of appointment, with waiting times significantly influenced by appointment timing (*χ*²_1_ = 290.88; *P*<.001). Cost distributions varied significantly by gender, age, service frequency, distance, and service type (all *P*<.001), with service type and distance identified as key cost determinants (*P*<.001). Patient satisfaction was consistently high across periods, with no significant difference (Mann-Whitney *U*=5,090,149; *P*=.38). Nurses expressed positive perceptions of the model.

**Conclusions:**

The internet hospital plus home nursing model effectively combines online diagnosis with in-home care, creating a closed-loop service that improves accessibility—particularly for older adults and mobility-impaired patients. Pilot implementations in Tianjin demonstrated benefits in convenience, accessibility, and cost-effectiveness, alongside high patient satisfaction and nursing staff approval. Given the aging population, this model holds significant potential for broader adoption. To ensure sustainable development, enhanced safety mechanisms and policy support are recommended. As an integrated health care model with Chinese characteristics, it also offers valuable insights for the global digital health sector.

## Introduction

The internet hospital model is a comprehensive medical service platform that uses network information technology to integrate the functions of consultation, prescription, payment, and drug delivery into a single platform using online follow-up and routine consultation [1]. Doctors at internet hospitals can effectively perform disease diagnosis and consultation and deliver medicines to patients’ homes through express delivery. However, patients who require nursing services such as pressure ulcer care, wound care, and venous blood collection are still required to visit a physical hospital after online consultation. Thus, home nursing services could potentially make up for the service shortcomings of internet hospitals. Internet + nursing service refers to the provision of nursing services by registered nurses of medical institutions using an “online application and offline service” approach for discharged patients, patients with mobility disabilities, and patients with chronic or common diseases using the Internet and other information technologies [2]. The internet plus nursing service is considered to be an economical and efficient supplement to physical hospital nursing services, resolving the time and space limitations of the traditional nursing model and integrating nurses’ fragmented time to provide nursing services [3,4]. In 2019, the China Health Commission formally released the “Notice on Pilot Work of INS,” which began the implementation of internet plus nursing service. Subsequently, nurses from public hospitals and community health centers in China have been encouraged to provide home nursing through the “nursing plus” cloud hospital platform, and patients can make online appointments for home nursing in Ningbo City, Zhejiang Province [5]. In December 2020, the “Notice on Further Promoting the Pilot Work of Internet plus Nursing Service” was issued. The internet plus nursing service model has been widely applied for specialty care, chronic disease management, and geriatric care [6]. In 2021, Wuhan Central Hospital released an “e-Home nursing” service, which was combined with community health service centers to provide home nursing care for discharged patients and patients with disabilities, advanced age, cancer, and unconsciousness through an online appointment system [7]. In addition to the case studies mentioned above, studies of internet plus nursing service have examined patient demand analysis [8,9], treatment effect comparison [10,11], nurse’s cognition and willingness [12,13], and comprehensive evaluation indices [14]. In the previous internet plus nursing service studies, the internet has served only as an information transmission channel and carrier for home nursing appointment, which cannot integrate online medical service by doctors or ensure the continuity of medical and nursing services. However, in the context of the rapid development of internet hospitals, patients with chronic or common diseases have received online diagnosis and treatment. Some internet hospital patients may also require online nursing guidance or home nursing after online consultation, leading to the development of the internet hospital plus home nursing. The internet hospital plus home nursing refers to integrating both doctors’ medical services and nurses’ care services using an internet platform, so patients can obtain integrated online and offline medical care services, which also facilitates service supply. However, few studies have examined the innovative internet hospital plus home nursing model.

Home-based care was developed relatively early in foreign countries and has been widely used in rehabilitation care, palliative care, geriatric care, maternal and child care, and wound care [15]. In the American Home Health Medical Care model, an interdisciplinary team is led by a physician or advanced practice nurse and provides longitudinal health care management (including comprehensive health assessment, multidisciplinary care coordination, and patient education) primarily for long-term household patients with chronic diseases and those aged 85 years and older. The American Home Health Care model is primarily delivered by registered nurses or caregivers who provide nursing care to patients with mild cases and people with disabilities through an appointment or governmental assessment process, and the service program requires the support of a physician for prescribing medications [16-18]. Japanese home nursing has been independently deployed by different levels of medical institutions, which formed professional teams of caregivers, therapists, and general practitioners to provide primary care, medical care, and living care for older people who are unable to take care of themselves because of disability, postoperative rehabilitation, or chronic disease [19]. The Hospital at Home department was established in Australian public hospitals using My Health Record to share medical information, enabling the senior nursing team to conveniently provide professional services such as injections, wound care, and health education to meet the needs of the older patients with chronic diseases, postoperative rehabilitation, and disabilities at home [20,21]. Studies related to home-based care in other countries have included disease care effect comparison [22,23], nursing method research [24,25], comprehensive evaluation [26], case promotion and application research [27], and nurse training [28]. Recent studies have also highlighted the growing integration of digital health platforms in home care services, particularly in the context of aging populations and postpandemic health care delivery [29-31]. However, home-based care in foreign countries is mainly carried out by physical hospitals, and no cases of internet hospital plus home nursing have been reported.

In the context of the rapid development of internet hospitals in China, and under the guidance of internet plus nursing service policy, the internet hospital plus home nursing has emerged as a new nursing model that connects online medical with offline nursing services. Therefore, this study aimed to evaluate the implementation outcomes of the internet hospital plus home nursing model by examining its convenience, accessibility, cost-effectiveness, patient satisfaction, and nurses’ perceptions. We hypothesized that internet hospital plus home nursing would demonstrate significant advantages in these aspects compared to traditional care models. A mixed-methods approach was used to test these hypotheses.

## Methods

### Research Subject

We examined the Internet Hospital of Tianjin Medical University General Hospital (TJMUGH) as the research objective in this study. This internet hospital was established in March 2020 to improve patients’ access to medical care and medication in the context of the prevention and control of the COVID-19 pandemic. However, patients who needed to provide blood, urine, and stool specimens, and those who required other nursing operations after online diagnosis and consultation via the internet hospital still needed to attend a physical hospital. To address the shortcomings of internet hospital services and patients’ needs for home nursing care, the internet hospital added home nursing services in November 2020 and established internet hospital plus home nursing as a new service mode. The internet hospital plus home nursing takes into account the service needs of patients in both internet hospitals and physical hospitals, adopts the service mode of online application in the internet hospital platform and offline home nursing by nurses at the patient’s home, and provides diversified nursing services. The internet hospital plus home nursing is divided into regular service, specialized service, and long-distance service. Regular service refers to a nurse scheduling to serve multiple patients. Specialized service refers to a nurse scheduling to serve a single patient. Both service types are applicable only when the service distance is less than 10 km. Long-distance service refers to a nurse scheduling to serve a single patient, with a distance of greater than 10 km and less than 100 km. The basic fees for these 3 service types are shown in Table 1. The fee for each internet hospital plus home nursing service item is charged separately, which is the same system used at physical hospitals.

**Table 1.** Basic fees for 3 types of internet hospital plus home nursing service (Fees are in Yuan; the average exchange rate during the study period was approximately US $1=Yuan 0.13975). Regular and specialized services are limited to service distances of 10 km or less, while only long-distance service is available for distances greater than 10 km.

Service type	Fees (Distance ≤3km)	Fees (3 km<Distance ≤ 5 km)	Fees (5 km<Distance ≤ 10 km)	Fees (10 km<Distance ≤ 30 km)	Fees (30 km<Distance ≤ 100 km)
Regular services	80	90	100	—^a^	—
Specialized services	140	160	180	—	—
Long distance services	—	—	—	300	450

a Not available.

At the internet hospital plus home nursing we examined, a total of 24 internet hospital plus home nursing service items are available, as follows: intravenous blood collection, urine and stool retention, vital signs monitoring, blood glucose monitoring, pressure injury care, wound care, hypodermic injections, peripherally inserted central catheter (PICC) maintenance, intramuscular injections, stoma care, health education, urinary catheterization, drainage bag replacement, peripheral intravenous needle maintenance, breastfeeding guidance, neonatal percutaneous bilirubin measurement, oxygen inhalation therapy, incontinence care guidance, indwelling catheterization care, specialty wound care (pacemaker wounds, perineal lateral incision wounds, bone traction one-stage healing wounds), extubation, oral care, incontinence dermatitis care, and stress injury prevention. For every internet hospital plus home nursing item, there are detailed instructions regarding appropriate patients, service content, precautions, cancelation of appointments, and refund mechanisms for patients’ understanding.

### Data Collection and Inclusion Criteria

#### Demand Side

**Figure 1.**
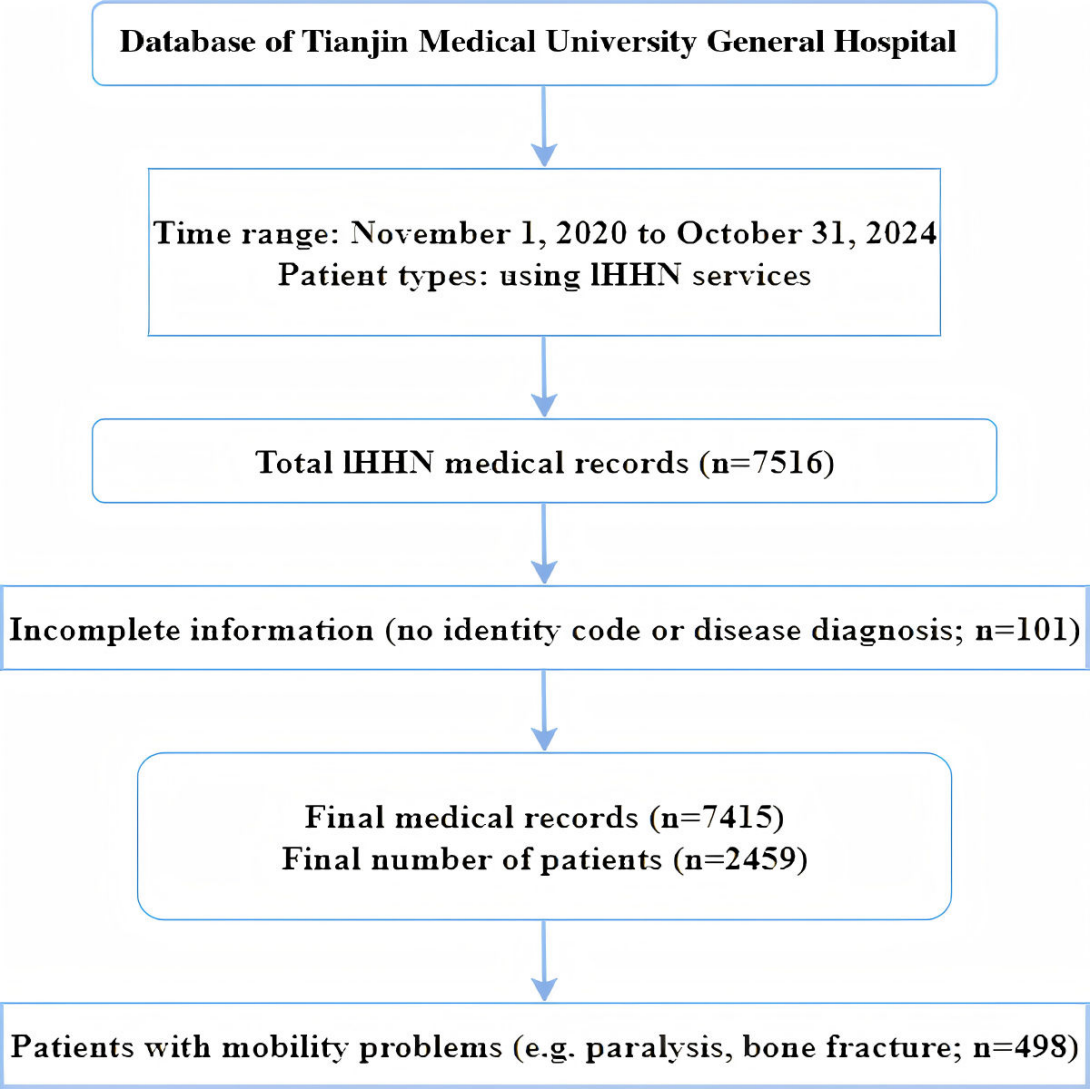
Patients’ data collection flowchart. IHHN: internet hospital plus home nursing.

We collected medical records of patients using data of internet hospital plus home nursing from November 1, 2020 to October 31, 2024 from the hospital database. A total of 7516 Internet hospital plus home nursing records were extracted, which included patients’ age, gender, home address, appointment time, service time, service item, service cost, major disease diagnosis, and comprehensive evaluation data. We excluded 101 records with incomplete identification information or disease diagnosis. Finally, 7415 records attributed to 2459 patients were included. There were 498 patients with mobility problems such as paralysis, bone fracture, leg disease, unconsciousness, and being bedridden (Figure 1).

#### Supply Side

Based on our literature review of existing internet + nursing service assessment tools [3,12-14]. We collected data on patients using internet hospital plus home nursing and conducted an online survey to assess nurses’ perceptions. The finalized questionnaire comprised 4 modules (see Multimedia Appendix 1): (1) nurse demographics and professional characteristics: including age, gender, department, years of work experience, professional title, and previous training in internet plus health nursing (internet hospital plus home nursing); (2) attitudes towards internet hospital plus home nursing: assessed via 10 items (eg, “Internet hospital plus home nursing has improved healthcare accessibility for patients with mobility impairments”); (3) self-assessed knowledge of internet hospital plus home nursing: assessed via 8 items (eg, “Safety protocols for home visits are adequately addressed”); and (4) qualitative feedback: Solicited through 3 open-ended questions (eg, “Key areas requiring improvement in internet hospital plus home nursing services”).

In December 2024, we conducted an anonymous online survey of nurses who had participated in the TJMUGH Nursing Department’s internet hospital plus home nursing services through the Questionnaire Star platform [32]. The Questionnaire Star platform is an online network service platform specializing in questionnaire surveys in China. A total of 107 valid questionnaires were collected.

#### Study Design

We collected data on patients using internet hospital plus home nursing and nurses’ perceptions. The internet hospital plus home nursing service flow was compared with the physical hospital nursing service to verify its convenience. The distribution of patients using internet hospital plus home nursing and the service waiting time were used to verify its accessibility. The basic internet hospital plus home nursing fee was compared with the cost of transferring patients by ambulance to a physical hospital, to validate its relative cost. Patients’ comprehensive evaluation scores were used to analyze the degree of patient satisfaction. A questionnaire survey was used to obtain nurses’ perceptions.

It is impossible to determine whether ambulance transfers were solely for nursing services. Cost estimates (comparing internet hospital plus home nursing costs with patient transfer costs) were based on a Geographic Information System-simulated route lengths and publicly available ambulance pricing schedules issued by the Tianjin Municipal Health Commission. Caution is needed in generalizing these results to other regions due to the heterogeneity of policies and resource allocation.

### Quantitative and Qualitative Methods

#### Number of Internet Hospital Plus Home Nursing Applications

We used the *χ*^2^ test to analyze the distribution of patients of different genders and ages in the number of internet hospital plus home nursing applications.

#### Patients' Major Disease Diagnosis Classification

Frequency tables were used to count the main disease diagnosis, geographical distribution, and service items of internet hospital plus home nursing patients. We mapped the geographic distribution of patients at the district level using ArcMap 10.8 software (Environmental Systems Research Institute) to show the patients’ regional distribution.

### Comparison of Patients’ Fees Between Internet Hospital Plus Home Nursing and Patients Transferring to Hospital

We examined the addresses and floors of 498 patients with limited mobility. A Python (Python Software Foundation) program was written to access the Amap Web Service API (Amap Software Co, Ltd) to simulate the transport routes for patients with limited mobility, and to calculate the corresponding distances and transfer costs. The details of the simulated cost of transferring patients to hospital and a preliminary sensitivity analysis using different fee assumptions are provided (see Multimedia Appendix 2). Given the skewed distribution of cost data and unequal variances between groups, the Mann-Whitney *U* test was applied to compare the cost differences between internet hospital plus home nursing and simulated transferring patients to the hospital across demographic and service types. Multivariate linear regression was used to explore associations between characteristic factors and costs.

### Patients' Degree of Satisfaction

From November 1, 2020 to October 31, 2021, internet hospital plus home nursing was evaluated by patients using 3 indicators: nurse professionalism, service punctuality, and service attentiveness, with 100 points for each, and the mean of the 3 indicator scores was the degree of satisfaction. To evaluate the degree of satisfaction with internet hospital plus home nursing in more detail, the evaluation index was modified on November 1, 2021. The degree of satisfaction with internet hospital plus home nursing was evaluated comprehensively using 5 indicators: service attitude, service skill, service price, service punctuality, and service experience, with 100 points for each, and the mean of 5 indicators scores was the degree of satisfaction. A *U* test was used to compare and analyze the degree of patient satisfaction during 2 time periods.

### Ethical Considerations

This study involved an analysis of patient data from the hospital database and nurse data from the survey; this did not involve human experimentation or compensation. The patient data used for this research were retrospectively extracted from the hospital information system and were fully anonymized, and the individual informed consent from patients was waived by the Ethics Committee. The nurse data comprised questionnaires answered by nurses who participated in the internet hospital plus home nursing with previous approval. All participating nurses were informed of the research purpose and voluntarily completed the survey anonymously. To ensure patient and nurse privacy and confidentiality, all identifiable personal information such as names, addresses, and contact details were removed during data extraction. All patient- and nurse-related datasets met ethical and legal requirements for data protection, and secondary use of these data was approved by the Ethics Committee. In addition, the research team complied with all relevant local, national, and international laws and regulations regarding the protection of personal information, privacy, and human rights. The Ethics Committee of the Tianjin Medical University General Hospital granted approval for this study following a thorough review of the research protocol (approval IRB2024-YX-066‐01).

## Results

**Figure 2.**
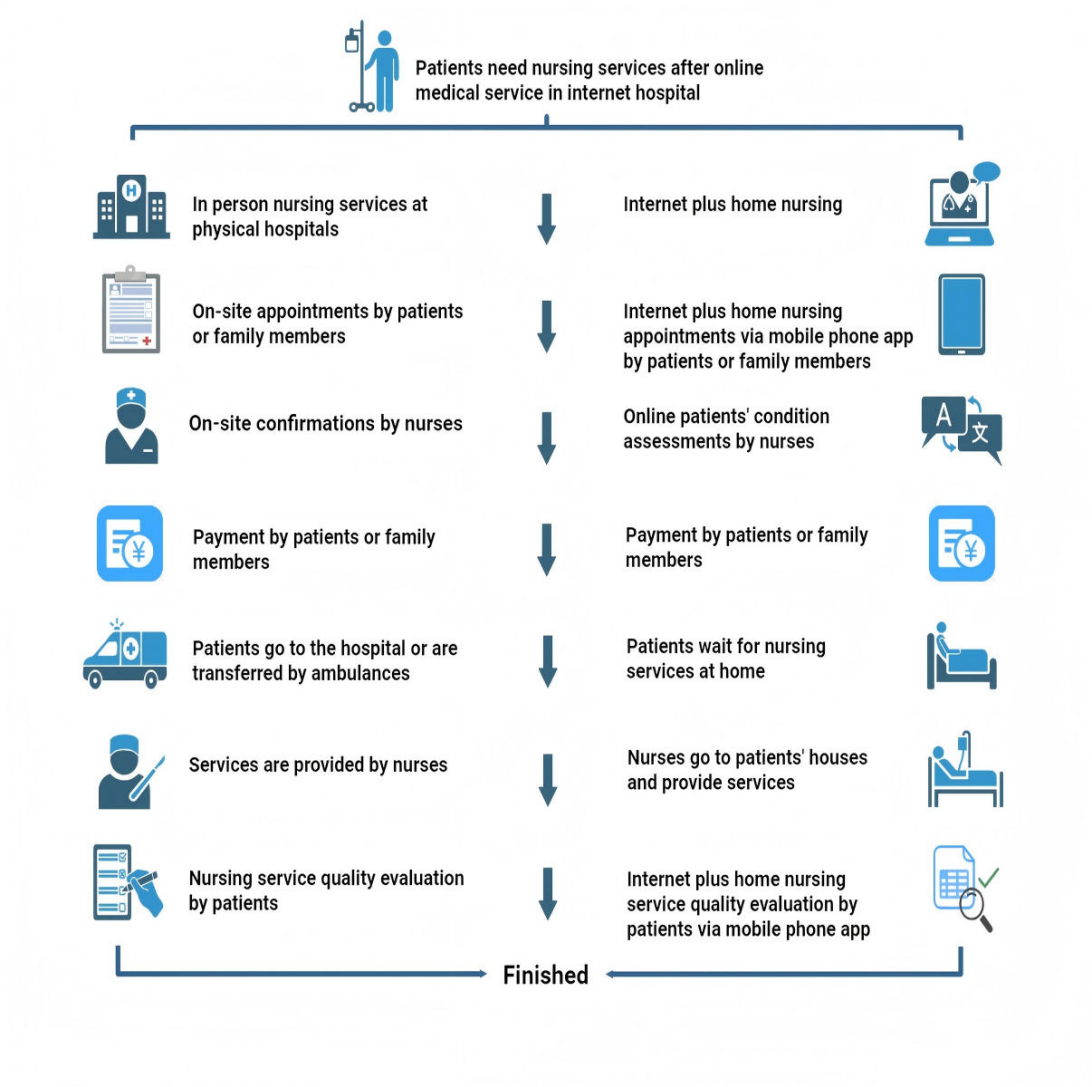
Flowchart of patients receiving nursing service in a physical hospital and an internet hospital plus home nursing.

Patients apply for home nursing services through the mobile phone app of the internet hospital plus home nursing (Create Medical Information Co, Ltd) after receiving medical service from the internet hospital, which eliminates the need to go back and forth to the physical hospital for making appointments, payment, and receiving service. Patients can conveniently obtain medical service and home nursing via internet hospitals plus home nursing at home (Figure 2).

### Demand Side

#### The Number of Internet Hospital Plus Home Nursing Applications

The results revealed no gender-related differences in the number of internet hospital plus home nursing applications (*P*=.54). However, we found a significant difference in the number of internet hospital plus home nursing applications between patient cohorts of different ages (*P*<.001). Among age groups, patients aged 60 years and older had the highest number of internet hospital plus home nursing applications (2120/2459, 86.2%). Patients aged 20 years and younger had the lowest number of internet hospital plus home nursing applications (8/2459, 0.3%) (Table 2).

**Table 2.** Distribution of patients using internet hospital plus home nursing by gender and age cohort (n=2459).

Characteristics	One time, n/N (%)	Two times, n/N (%)	≥ Three times, n/N (%)	Chi-square (*df*)	*P* value
Sex				1.23 (1)	.54
Female	738/1350 (54.7)	235/1350 (17.4)	397/1350 (29.4)
Male	569/1109 (51.3)	182/1109 (16.4)	338/1109 (30.5)
Age (y)				29.86 (4)	<.001
≤20	2/8 (25.0)	1/8 (12.5)	5/8 (62.5)		
20<age ≤ 40	105/151 (69.5)	24/151 (15.9)	22/151 (14.6)
40<age ≤ 60	100/180 (55.0)	28/180 (15.6)	52/180 (28.9)
60<age ≤ 80	607/1214 (50.0)	214/1214 (17.6)	393/1214 (32.4)
>80	493/906 (54.4)	150/906 (16.6)	263/906 (29.0)

### Patients’ Major Disease Diagnosis Classification

Patients with a major oncological disease diagnosis accounted for the largest proportion of internet hospital plus home nursing applications (1468/7415, 19.8%), while those with cardiovascular disease diagnosis (876/7415, 11.8%) and neurological disease diagnosis (747/7415, 10.1%) had the second and third highest number of internet hospital plus home nursing applications, respectively (Table 3).

**Table 3.** The top 10 major disease diagnoses of patients receiving internet hospital plus home nursing services.

Major diagnosis	Applications, n (%)	Patients, n (%)
Oncological diseases	1468 (19.8)	415 (16.9)
Cardiovascular diseases	876 (11.8)	416 (16.9)
Neurological diseases	747 (10.1)	350 (14.2)
Metabolic diseases	665 (9)	129 (5.3)
Hematologic diseases	597 (8.1)	177 (7.2)
Autoimmune diseases	407 (5.5)	167 (6.8)
Respiratory diseases	360 (5.5)	74 (3)
Dermatological diseases	358 (4.8)	96 (3.9)
Genitourinary diseases	218 (2.9)	103 (4.2)
Renal diseases	187 (2.5)	95 (3.9)

#### The Number of Internet Hospital Plus Home Nursing Service Items

Table 4 shows that intravenous blood collection was the service that was most frequently applied for (4899/7415, 66.1%), followed by PICC maintenance (927/7415, 12.5%) and urinary catheterization (506/7415, 6.8%).

**Table 4.** Top 10 service items of patients receiving internet hospital plus home nursing services.

Service items	Applications, n (%)	Patients, n (%)
Intravenous blood collection	4899 (66.1)	1919 (78)
PICC^a^ maintenance	927 (12.5)	164 (6.7)
Urinary catheterization	506 (6.8)	161 (6.6)
Pressure injury care	322 (4.3)	55 (2.2)
Wound care	181 (2.4)	70 (2.9)
Peripheral intravenous needle maintenance	140 (1.9)	50 (2)
Indwelling catheterization care	71 (1)	6 (0.24)
Stoma care	63 (0.9)	21 (0.9)
Specialized wound care	43 (0.6)	18 (0.7)
Intramuscular injection	38 (0.5)	12 (0.5)

a PICC: peripherally inserted central catheter.

### Patients’ Geographic Distribution

**Figure 3.**
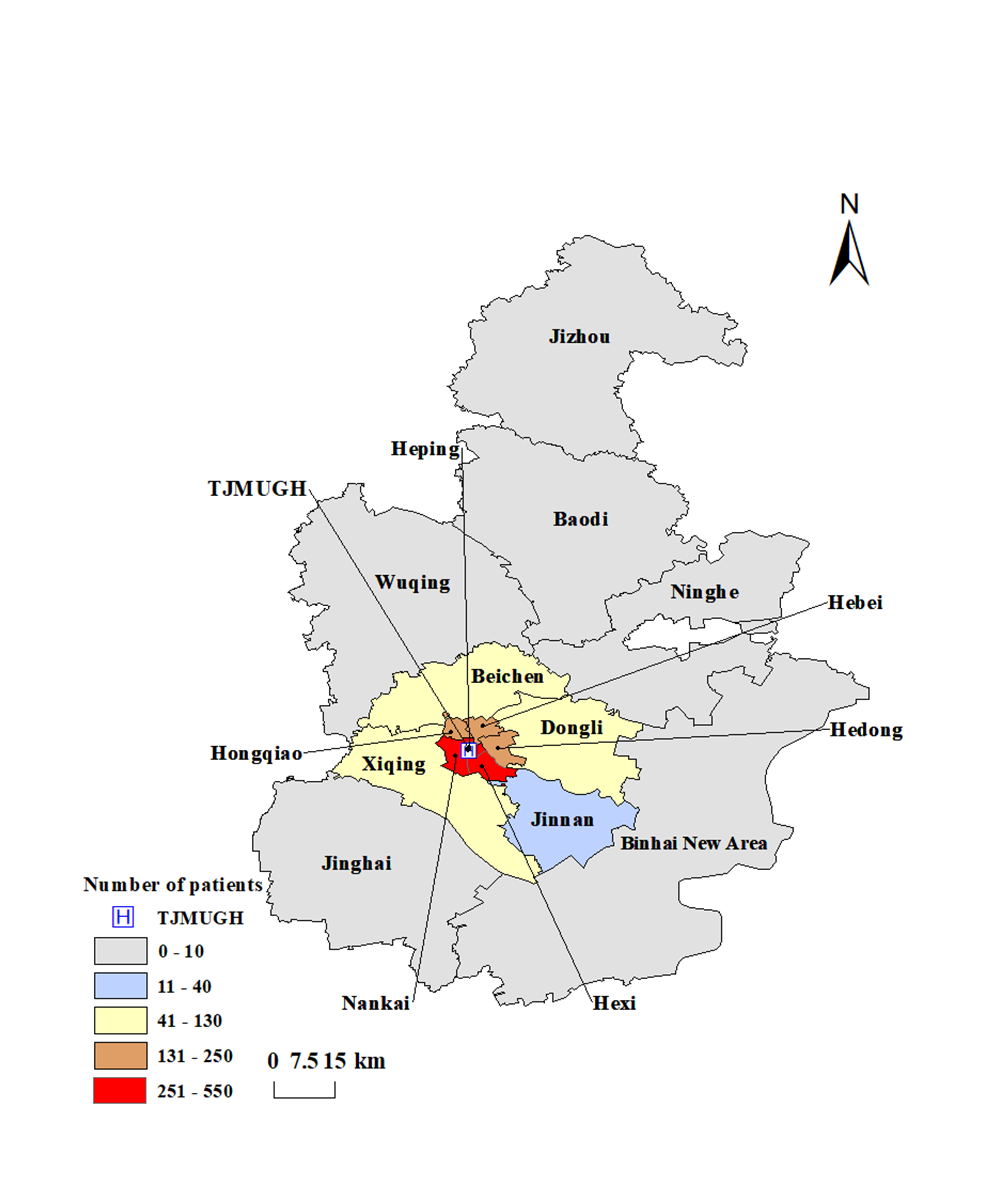
District-level geographic distribution of patients. TJMUGH: Tianjin Medical University General Hospital.

Patients using internet hospital plus home nursing services were mainly concentrated in the Nankai, Hexi, Heping, Hedong, Hebei, and Hongqiao districts (2119/2459, 86.2%). Of these regions, Nankai district accounted for the largest number of internet hospital plus home nursing patients (524/2459, 21.3%), and the largest number of times internet hospital plus home nursing was used (1739/7415, 23.5%). Jinnan, Binhai, Jinghai, and Jizhou districts were further away from TJMUGH and had fewer patients (Table 5). The regional distribution map of patients showed that the patients’ locations were mainly centered around TJMUGH (Figure 3).

**Table 5.** District-level geographical distribution of patients receiving internet hospital plus home nursing services.

District of Tianjin City	Patients, n (%)	Applications, n (%)	Applications per person, mean (SD)
Nankai District	524 (21.3)	1739 (23.5)	3.32 (0.68)
Hexi District	487 (19.8)	1439 (19.4)	2.95 (0.68)
Heping District	407 (16.6)	1002 (13.5)	2.46 (0.68)
Hongqiao District	241 (9.8)	592 (8)	2.46 (0.68)
Hedong District	232 (9.4)	809 (10.9)	3.49 (0.68)
Hebei District	228 (9.3)	712 (9.6)	3.12 (0.68)
Xiqing District	128 (5.2)	535 (7.2)	4.18 (0.68)
Dongli District	90 (3.7)	183 (2.5)	2.03 (0.68)
Beichen District	76 (3.1)	277 (3.7)	3.64 (0.68)
Jinnan District	36 (1.5)	104 (1.4)	2.89 (0.68)
Binhai New Area	7 (0.3)	13 (0.2)	1.86 (0.68)
Jinghai District	3 (0.1)	10 (0.1)	3.33 (0.68)

### Patients’ Appointment Time and Service Waiting Time

All patients had access to services within 1‐2 days after making their internet hospital plus home nursing service appointments. All patients were able to access services within one to two days after making appointments through the Internet hospital plus home nursing service. Significant differences in waiting times were observed across different appointment time slots (*χ*²_1_=290.88; *P*<.001). Among patients who made appointments between 6 AM and 12 PM, 64.9% (1873/2885) experienced a 1-day waiting period, while 44.6% (2022/4530) waited for 2 days. In contrast, for appointments made between 12 PM and 6 PM, 35.1% (1012/2885) of patients waited for a day, whereas 55.4% (2508/4530) waited for 2 days.

### Comparison of Patients’ Fees Between Internet Hospital Plus Home Nursing and Patients Transferring to a Hospital

Across 498 patients with limited mobility, simulation results showed significantly lower costs for the internet hospital plus home nursing services compared with ambulance transfers to physical hospitals in all demographic and service subgroups. The Mann-Whitney *U* test indicated statistically significant differences (*P*<.001) across gender, age groups, number of visits, service distances, and service types (see Table S1 in Multimedia Appendix 3). Even in long-distance services, the internet hospital plus home nursing services remained more cost-effective (*P*=.007). To further explore fee-influencing factors, multivariate linear regression was performed (see Table S2 in Multimedia Appendix 3). Service distance and service type emerged as significant predictors of cost differences (*P*<.001). Internet hospital plus home nursing’s specialized and long-distance services were associated with higher costs but still showed a net saving compared to hospital transfer. A sensitivity analysis (see Table S5 in Multimedia Appendix 2) using different fee assumptions confirmed the robustness of these results. Internet hospital plus home nursing maintained economic advantage under all modeled scenarios.

### Patients’ Degree of Satisfaction

As of October 31, 2024, a total of 6980 patients responded to an anonymous satisfaction survey regarding services provided through the internet hospital plus home nursing mobile app (response rate: 94.1%, 6980/7415). Patient satisfaction scores remained high across both assessment periods. Specifically, during the period from November 1, 2020, to October 31, 2021, 2068 patients (29.6%) reported a mean satisfaction score of 99.80 (SD 2.65). In the subsequent period from November 1, 2021, to October 31, 2024, 4912 patients (70.4%) reported a mean satisfaction score of 99.89 (SD 1.50). A Mann‑Whitney *U* test indicated no statistically significant difference in satisfaction scores between the two periods (U=5,090,149.00, *P*=.38).

### Nurses’ Perceptions

We distributed 120 questionnaires to nurses who had engaged in internet hospital plus home nursing services, and 107 were returned (recovery rate 89.2%). Nurses’ basic information and the results of the questionnaire survey are provided in Multimedia Appendix 1. In summary, nurses supported internet hospital plus home nursing (93/107, 86.9%), and their attitudes toward internet hospital plus home nursing were positive. Specialized training of internet hospital plus home nursing was necessary (88/107, 82.2%). Detailed knowledge of the patient’s medical and allergy history was very important (97/107, 90.7%). The key factors affecting internet hospital plus home nursing quality were the professional skill and experience of nurses (91/107, 85.1%). The main factor affecting the safety of patients was sudden illness deterioration without timely medical team support (97/107, 90.7%). The main factor affecting the safety of nurses was the violent behavior of the patients or their family members (93/107, 86.9%). It is very necessary to equip a video recorder and record the service process (100/107, 93.5%) as well as to have a special person to accompany nurses (92/107, 86%) . It needs to clarify price standards and insurance reimbursement policies (98/107, 91.6%). In the future, it is expected that specialized nursing care (88/107, 82.2%), rehabilitation training (84/107, 78.5%) as well as a comprehensive internet hospital plus home nursing information system (90/107, 84.1%) should be provided.

## Discussion

### Principal Findings

This mixed-methods study demonstrates that the internet hospital plus home nursing model effectively delivers convenient, accessible, and cost-effective home-based care, particularly for older adults and mobility-impaired patients. Key implementation outcomes include high patient satisfaction, positive nurse perceptions, significant cost savings compared to traditional ambulance transfers, and efficient service delivery within 1‐2 days.

This mixed-methods study evaluated the implementation outcomes of the innovative internet hospital plus home nursing model. The primary findings indicate that the internet hospital plus home nursing model effectively provides convenient, accessible, and relatively cost-effective home nursing services, particularly benefiting older adults and mobility-impaired patients. The key findings include: high patient satisfaction scores (average scores of 99.80/100 and 99.89/100 across two periods); nurses maintained a positive attitude, with a support rate of 86.9%; compared with the cost of ambulance transportation, accessing internet hospital plus home nursing routine and specialized nursing services incurred significantly lower costs; and service delivery was efficient, typically completed within 1 to 2 days. Cancer patients constituted the primary user group, and venous blood collection was the most frequently requested service. Patient geographic distribution was concentrated primarily in areas adjacent to physical hospitals.

Internet hospital plus home nursing expands the accessibility of treatment to a broader group of patients. Internet hospitals have extended the service radius of physical hospitals, and an increasing number of patients are choosing internet hospital-based medical services [33]. Recent evidence supports the effectiveness of such integrated models in improving health care access and patient outcomes, especially in remote and underserved areas [34,35]. Internet plus nursing service is also widely accepted by patients and nurses and has been reported to effectively resolve the problem of nursing care access for mobility-impaired patients [8,12]. Internet hospital plus home nursing expands the method of delivering medical and nursing care services, optimizing online and offline medical and nursing care service on the basis of the internet hospital information platform, and providing integrated medical and nursing service to all patients. The current results suggest that patients preferred the internet hospital plus home nursing, with many patients (735/2459, 29.9%) using it more than 3 times. Of the various districts, patients in Xiqing District had the highest number of applications (mean 4.18 times, SD 0.68). The internet hospital plus home nursing mobile app and the promise of service within 2 days improved service accessibility. As population aging progresses, the demand for internet nursing services is gradually increasing over time [9], and the population of internet hospital plus home nursing patients is likely to expand.

The internet hospital plus home nursing model can reduce the cost of treating diseases. Internet hospitals are widely considered to overcome the limitations of time and space [36]. Internet hospital plus home nursing can not only overcome time and space constraints, but can also reduce the cost of transportation, time, and accompanying personnel. The economic advantage is obvious for patients who choose internet hospital plus home nursing routine services and dedicated services. The cost of long-distance service is similar to that of transferring patients to hospital by ambulance. Long-distance service involves increased transportation costs but still saves patients and their family members indirect costs such as time and companionship. At present, the number of patients receiving internet hospital plus home nursing long-distance service is small, which also contributes to its high cost. In the future, long-distance service cost will gradually decrease when the number of applications increases and economies of scale can be achieved. Long-distance service is relatively economical once economies of scale can be obtained compared with the cost of ambulance transfer of one patient in a single trip.

Although long-distance internet hospital plus home nursing services (10‐100 km) have demonstrated strong cost-effectiveness compared to traditional ambulance transfers, their uptake in remote districts remains extremely limited. For instance, only 13 patients from Binhai New Area were recorded among 7415 cases (0.2%). This underuse may stem from multiple barriers. Logistically, long-distance visits require greater scheduling flexibility, higher vehicle idle times, and increased staff input, reducing operational efficiency. Economically, current medical insurance reimbursement policies may inadequately compensate providers for the extra resources needed, disincentivizing participation. Institutionally, the lack of cross-district service coordination or standardized referral pathways poses regulatory challenges. Patients in remote regions may also lack awareness or trust in internet hospital plus home nursing services, perceiving them as less reliable than traditional emergency transport, especially for urgent needs. Additionally, digital access gaps or low e-health literacy may hinder online scheduling. To address these constraints, we recommend policy incentives such as distance-based subsidies, intelligent route optimization to improve efficiency, targeted public education campaigns, and integration of telemedicine for hybrid care delivery. These measures could improve both the accessibility and scalability of internet hospital plus home nursing services in geographically underserved populations.

Internet hospital plus home nursing was supported by both patients and nurses. Internet hospital plus home nursing is a patient-centered service using a model that is similar to private, customized professional medical and nursing services. Nurses who carry out internet hospital plus home nursing have rich professional knowledge and clinical experience. Before working in internet hospital plus home nursing, these nurses needed to receive specialized training to cope with emergencies such as additional requests, verbal threats, and violence from patients or family members in the process of home nursing. A previous study reported that specialized training contributes to the improvement of patient satisfaction [28]. In the actual service process, nurses need to comprehensively assess the patient’s detailed medical history and allergies to determine whether the patient is suitable for internet hospital plus home nursing, then develop personalized care plans for patients with different types of diseases. Patients can obtain nursing services at home that are of the same quality as hospital-based nursing services, which greatly improves their experience and satisfaction. In this study, patients’ comprehensive evaluation results indicated that patients consistently had a high level of satisfaction with internet hospital plus home nursing services. Nurses maintain a positive perception toward internet hospital plus home nursing services. However, attention needs to be paid to nurses’ professional competence and experience, and multifunction information systems are necessary to ensure the quality and safety of internet hospital plus home nursing services.

### Comparison With Previous Work

Internet hospital plus home nursing is designed to bridge the gap between medical and nursing services. Internet nursing service platforms that only recruit nurses to provide home nursing services were previously reported to have low reliability and standardization [6]. The internet hospital examined in this study relies on a brick-and-mortar hospital and is able to provide patients with online consultation and diagnosis, prescriptions of medicines, examinations, tests, and nursing services. At this internet hospital, it is also possible to transfer patients with serious conditions to the outpatient department, emergency department, or inpatient department of a physical hospital for further treatment. However, patients with chronic diseases such as diseases of oncology, cardiovascular, neurology, or mobility problems, who only require home nursing after an online medical service do not have to go to a physical hospital. Thus, internet hospitals need home nursing services such as intravenous blood collection, PICC maintenance, and urinary catheterization to make up for the limitations of their care provision. As nurses do not have the ability to prescribe medications independently, doctors’ prescriptions are still needed when patients are treated by nurses. Therefore, internet hospital plus home nursing services can effectively make up for the shortcomings of both internet hospitals and internet nursing services, achieving all-round services of online consultation, diagnosis, prescription and offline medication delivery, home nursing, and patient referral. Furthermore, internet hospital plus home nursing not only ensures the continuity of medical and nursing services, but also makes patients’ treatment more convenient.

### Positioning Internet Hospital Plus Home Nursing Within Global Home-Based Care Models

China’s internet hospital combined with internet hospital plus home nursing model aligns with international home health care models such as the “Hospital-to-Home” projects widely implemented in countries like the United States, Japan, and Australia, sharing the core objective of providing home-based medical care services to patients [15-21]. In terms of reducing barriers to accessing medical services for vulnerable groups such as the older adults and those with mobility impairments, conducting routine technical services such as venous blood collection and maintenance of PICC, and improving patient satisfaction [15,18,22,23], these models have demonstrated similar outcomes.

However, the internet hospital plus home nursing model, which relies on China’s “internet hospital” system, has developed distinctive features that differentiate it from international practices. This model centers on a dedicated digital platform, establishing a seamless “Online-to-Offline” integration process and a “medical-nursing” collaboration mechanism that bridges “online medical consultation and diagnosis” with “offline home care services,” overcoming the administrative limitations of traditional physical hospitals or community health systems in resource allocation and service extension [3]. Compared to the US model, which emphasizes doctor-led multidisciplinary teams [16], and the Australian model, which emphasizes the application of shared electronic health records [20], the innovation of internet hospital plus home nursing services lies in using internet hospitals as central coordination hubs, achieving systematic management of the entire service process through a unified digital entry point, thereby forming a unique new model variant in the global digital health field. Although it still faces challenges in terms of geographical accessibility in remote areas, its innovative architecture deeply integrated with the internet hospital system provides a reference-worthy family healthcare paradigm for regions where policies support the development of internet hospitals.

### Strengths and Limitations

To our knowledge, this study is the first to systematically characterize the innovative approach of internet hospital plus home nursing services in implementing the internet nursing service policy. We evaluated the implementation outcomes from multiple perspectives, and the findings provide practical evidence for optimizing internet hospital plus home nursing services.

First, this study used a single-center design based at the General Hospital of Tianjin Medical University, a tertiary-level A-class (top-tier) medical institution located within an economically developed municipality. All data were sourced from this institution. Although the comprehensive hospital network encompassed the entire city of Tianjin, patient uptake of remote services remained limited. Furthermore, the single-center design, situated in an urban setting, limits the generalizability of the results. Regional variations, particularly in the following three domains, may substantially affect the scalability of the comprehensive hospital network model: (1) Infrastructure disparities: rural and remote areas may encounter challenges including insufficient network coverage and low rates of mobile device ownership and penetration, hindering the implementation and dissemination of internet hospital plus home nursing services. (2) Human resource constraints: regions with limited resources often experience shortages in nursing staff and incomplete professional skill sets among existing personnel. (3) Population density: in regions with low population density, nurses’ travel time and associated costs are significantly higher compared to the urban study setting, potentially compromising the model’s economic feasibility.

Second, this design inherently limits the generalizability of the study results to diverse socioeconomic contexts or health care resource distribution environments (eg, rural areas or secondary hospitals), thereby significantly compromising external validity. Specifically: (1) The findings of the cost-benefit analysis, particularly the comparison between comprehensive hospital costs and patient transportation costs, are constrained by regional variations in medical infrastructure, population density, and regulatory frameworks. These variations include differing ambulance fees, nursing service pricing structures, and insurance reimbursement policies, limiting broader applicability; (2) the absence of a control group consisting of patients receiving traditional care services; and (3) a high proportion of repeat users (29.9% of patients received care ≥3 times), indicating potential self-selection bias. This bias suggests the sample may overrepresent patients with greater health care engagement or higher satisfaction levels, potentially leading to an overestimation of satisfaction metrics and nurse acceptance rates. These factors collectively constrain the external validity of the cost-effectiveness and satisfaction outcomes.

Third, this study exhibits significant methodological limitations in internet hospital plus home nursing patient satisfaction assessment: (1) Scale inconsistency: arbitrary expansion from 3 domains (professionalism, punctuality, and attentiveness) to 5 domains (attitude, skills, price, punctuality, and experience) compromised temporal comparability despite nonsignificant interperiod score differences (*P*=.38). Given the retrospective design of this study, although satisfaction evaluation indicators were modified, the scoring methodology retained a percentage-based framework. Comparative analysis of total satisfaction scores demonstrated that patient satisfaction with internet hospital plus home nursing services persisted at consistently high levels following indicator adjustments. (2) Inadequate content validity: omission of critical indicators (emotional support, communication clarity, and patient-physician trust) and qualitative mechanisms precluded identification of drivers for near-ceiling satisfaction (mean>99/100) or dissatisfaction sources. (3) The exceptionally high satisfaction scores (mean>99/100) warrant cautious interpretation. They primarily signify strong patient recognition of the internet hospital plus home nursing model’s core value proposition, specifically service accessibility and convenience, alongside fundamental trust in nursing staff professionalism. This is corroborated by a high service reuse rate (29.9% used ≥3 times) and positive nurse feedback. Crucially, however, these near-ceiling values may be inflated by the assessment tool’s limited sensitivity to high-end variation and potential social desirability bias. Consequently, they should not be interpreted as indicative of optimal service experiences across all microlevel dimensions and may mask potential improvement areas or unarticulated negative experiences (eg, safety concerns documented in nurse questionnaires but absent from patient ratings). In summary, these results robustly indicate the model’s broad patient acceptance and success in meeting core needs, rather than definitive evidence of flawless execution.

### Future Work

Internet hospital plus home nursing urgently requires the establishment of a comprehensive risk management framework. This framework must systematically address the core challenges of patient clinical safety and nurse practice safety. A dynamic risk assessment mechanism should be integrated, using standardized tools to evaluate key domains including patient medical stability, home environment safety, sociopsychological factors, and geographical accessibility [37]. Based on these assessments, a 3-tier risk stratification system (low, medium, and high) must be implemented, with corresponding safety protocols developed accordingly. A primary task is the clear delineation of the internet hospital plus home nursing service boundaries and responsibility allocation. Patient requirements exceeding the assessed scope of internet hospital plus home nursing services necessitate prompt referral to physical hospitals to ensure patient safety and service quality. Concurrently, nurse safety must be prioritized through multifaceted measures: (1) Technological measures: provision of personal alarm devices, authorized remote monitoring equipment, and real-time communication; (2) operational measures: establishment of standardized emergency response protocols covering scenarios of patient medical deterioration and safety threats; (3) legal safeguards: clear definition of authority boundaries and patient (or family) safety obligations [38], supplemented by specialized occupational insurance and postincident support mechanisms (including confidential reporting channels, and psychological and legal assistance); and (4) capability enhancement: Strengthened safety awareness training, conflict de-escalation techniques, basic self-defense instruction, and provision of protective equipment (such as personal alarms and recording devices) [39]. This systematic framework aims to enhance service transparency and traceability, providing objective evidence for dispute resolution [40]. It constitutes an essential prerequisite for optimizing home health care management, ensuring the safety of all stakeholders, and improving nurse engagement.

The internet hospital plus home nursing model is innovative, with no similar cases reported globally to date; consequently, no multicenter studies have been conducted. To enhance the external validity and validate the universality of the internet hospital plus home nursing model, subsequent studies will use a prospective, multicenter-controlled design. Research subcenters will be established in geographically diverse regions (encompassing varying socioeconomic levels, differential access to medical resources, and distinct policy environments). Through parallel comparison of the internet hospital plus home nursing model and the traditional inpatient care model, the study aims to elucidate the causal effects of its interventions and rigorously control for selection bias. Particular emphasis will be placed on regional variations in the cost-effectiveness analysis of the internet hospital plus home nursing model. To optimize sample representativeness and mitigate bias in satisfaction assessments, a stratified sampling strategy will be implemented to ensure adequate representation of key subgroups, including first-time service users, low-frequency users, and discontinued care recipients. This study demonstrates that the internet hospital plus home nursing model confers significant advantages. As the Tianjin Internet Medical Quality Control Center, TJMUGH will facilitate the implementation of this model across Tianjin and nationwide. The increasing adoption of the internet hospital plus home nursing model by medical institutions will establish the foundation for subsequent multicenter studies and facilitate future research.

Regarding patient satisfaction assessment, this study is a retrospective study, and the data used truly reflects patients’ evaluations of internet hospital plus home nursing services. Future research will establish a standardized core assessment instrument encompassing key dimensions (eg, emotional support, clarity of information communication, and physician-patient trust), ensuring the scales possess robust content validity and longitudinal comparability. The study will adopt a mixed-methods approach, integrating quantitative analysis with qualitative research to comprehensively explore the drivers of satisfaction and identify the root causes of potential dissatisfaction, thereby addressing the limitations inherent in reliance on single-method quantitative tools. To control for social desirability bias and response bias, the assessment process will use anonymized evaluations and incorporate third-party interviews to validate the authenticity of patient feedback.

### Conclusions

This study comprehensively analyzed the effect of internet hospital plus home nursing service as an innovative model of internet plus nursing service, demonstrating characteristics of convenience, accessibility, and relative cost-effectiveness within the Tianjin setting. The internet hospital plus home nursing service can resolve some of the shortcomings of online medical and nursing services, improving access to medical and nursing care for older adults and mobility-impaired patients and has high patient satisfaction and nurses’ positive perceptions. As population aging progresses, Internet hospital plus home nursing services are likely to become increasingly widespread. It is important to optimize these services from the perspective of ensuring patient treatment safety and nurses’ personal safety, and corresponding policy support. Furthermore, the core innovative contribution of internet hospital plus home nursing services resides in its capacity to establish a closed-loop system integrating “online diagnosis and treatment services” with “home care” via the internet hospital platform, thereby bridging the service gap between conventional internet health care and standalone home care. This service offers a novel integrated medical care approach with distinctive Chinese characteristics to the global digital health domain.

## Supplementary material

10.2196/76761 Multimedia Appendix 1 We conclude nurses' basic information and questionnaire survey results.

10.2196/76761 Multimedia Appendix 2 Cost comparison between internet hospital and home nursing and transferring patients to hospital using geographic information system (GIS)-based simulation technology and conduct a sensitivity analysis using different fees.

10.2196/76761 Multimedia Appendix 3 We compared the costs of the traditional hospital referral model with those of the integrated network hospital plus home care model.

## Abbreviations

PICC: peripherally inserted central catheter
TJMUGH: Tianjin Medical University General Hospital
