# Supplementary material for: An Internet Hospital Plus Home Nursing Model for Chronic Disease Patients: Mixed-Methods Study in Tianjin, China
Source: JMIR Nurs. 2025 Nov 5;8:e76761. doi: 10.2196/76761 (PMC12588391; doi:10.2196/76761)
Supplement: Multimedia Appendix 1 [file nursing-v8-e76761-s001.docx]

**Attachment 1**

**Nurses’ basic information and questionnaire survey results**

| Items | Number, n/N (%) |  |
| --- | --- | --- |
| **Section 1 Nurses’ basic information** |  |  |
| **1.Age(years)** |  |  |
| 20-25 | 9/107 (8.4) |  |
| 26-30 | 12/107 (11.2) |  |
| 31-35 | 19/107 (17.8) |  |
| 36-40 | 38/107 (35.5) |  |
| 41-60 | 29/107 (27.1) |  |
| **2.Gender** |  |  |
| Female | 6 /107(5.6) |  |
| Male | 101 /107(94.4) |  |
| **3.Departments (top10)** |  |  |
| General surgery | 44 /107 (41.1) |  |
| Orthopaedics | 20 /107 (18.7) |  |
| Urology | 12 /107 (11.2) |  |
| Internal medicine | 6 /107 (5.6) |  |
| Emergency Medicine | 5 /107 (4.7) |  |
| Rheumatology and Immunology | 4 /107(3.7) |  |
| Neurosurgery | 3 /107 (2.8) |  |
| Medical and Health Care | 2 /107 (1.9) |  |
| Pediatrics | 2 /107 (1.9) |  |
| Gynecology | 2 /107 (1.9) |  |
| **4.Degree** |  |  |
| College | 2 /107(1.9) |  |
| Undergraduate | 101/107 (94.4) |  |
| Graduate | 4 /107(3.7) |  |
| **5.Work experience (years)** |  |  |
| 1-5 | 17 /107(15.9) |  |
| 6-10 | 14/107 (13.1) |  |
| 11-15 | 34 /107(31.8) |  |
| 16-20 | 22/107 (20.6) |  |
| ＞20 | 20/107 (18.7) |  |
| **6.Job title** |  |  |
| Junior Nurse | 5 /107(4.7) |  |
| Supervisor Nurse | 21 /107(19.6) |  |
| Deputy chief nurse | 12 /107(11.2) |  |
| Chief nurse | 69 /107(64.5) |  |
| **Section 2:Attitudes Toward Internet hospital plus home nursing** | |  |
| **7.Which is your attitude toward providing Internet hospital plus home nursing?(SCQ)** | |  |
| A. **Strongly support.** | | 73 /107(68.2) |
| B. Support. | | 20 /107(18.7) |
| C. Neutral. | | 9 /107(8.4) |
| D.Somewhat oppose. | | 5 /107(4.7) |
| E. Strongly oppose. | | 0 /107(0.0) |
| **8. Which is patients’ attitude toward Internet hospital plus home nursing according to your experience?** **(SCQ)** | |  |
| A. Strongly support. | | 67 /107(62.6) |
| B. Support. | | 30 /107(28.0) |
| C. Neutral. | | 10 /107(9.4) |
| D. Somewhat oppose. | | 0 /107(0.0) |
| E. Strongly oppose. | | 0 /107(0.0) |
| **Section 3: Perceptions of Internet hospital plus home nursing** | |  |
| **9.Is it necessary to specially train Internet hospital plus home nursing for nurses?(SCQ)** | |  |
| A.Extremely necessary | | 62 /107(57.9) |
| B.Necessary | | 26 /107(24.3) |
| C.Neutral | | 14 /107(13.1) |
| D.Somewhat unnecessary | | 3 /107(2.8) |
| E.Completely unnecessary | | 2 /107(1.9) |
| **10.Is it necessary to use video recording devices during Internet hospital plus home nursing?(SCQ)** | |  |
| A.Extremely necessary | | 80 /107(74.8) |
| B.Necessary | | 20 /107(18.7) |
| C.Neutral | | 4 /107(3.7) |
| D.Somewhat unnecessary | | 2 /107(1.9) |
| E.Completely unnecessary | | 1 /107(0.9) |
| **11.Is it necessary to have a dedicated staff to accompany nurses during Internet hospital plus home nursing ?(SCQ)** | |  |
| A.Extremely necessary | | 74 /107(69.2) |
| B.Necessary | | 18 /107(16.8) |
| C.Neutral | | 10 /107(9.4) |
| D.Somewhat unnecessary | | 4 /107(3.7) |
| E.Completely unnecessary | | 1 /107(0.9) |
| **12.In your opinion,what is the primary benefit of Internet hospital plus home nursing for patients?(MSQ)** | |  |
| A. Delivers personalized and patient-centered nursing care. | | 97 /107(90.7) |
| B. Promotes fast recovery in a familiar home environment. | | 74 /107(69.2) |
| C. Improves patient and caregiver health literacy. | | 75 /107(70.1) |
| D. Reduces hospital re-admissions and healthcare costs. | | 86 /107(80.4) |
| E. Enhances accessibility for home bound or immobile patients. | | 100 /107(93.5) |
| **13.Before Internet hospital plus home nursing, what information is critical?(MSQ)** | |  |
| A. Patient’s detailed medical history and allergies. | | 97 /107(90.7) |
| B. Patient’s home environment and facilities. | | 79 /107(73.8) |
| C. Transportation and travel time to the patient’s home. | | 80 /107(74.8) |
| D. Patient and family background information. | | 70 /107(65.4) |
| E. Availability of nearby medical resources. | | 62 /107(57.9) |
| **14. What is the challenging aspect of communicating with patients and family member during Internet hospital plus home nursing?(MSQ)** | |  |
| A. Explaining medical procedures and knowledge. | | 63 /107(58.9) |
| B. Managing emotional distress. | | 69 /107(64.5) |
| C. Addressing additional requests. | | 83 /107(77.6) |
| D. Handing excessive family member interference. | | 75 /107(70.1) |
| E. Dealing with aggressive behavior. | | 74 /107(69.2) |
| **15. What is the key factor affecting the quality of Internet hospital plus home nursing?(MSQ)** | |  |
| A. Professional skills and experience. | | 91 /107(85.1) |
| B. Adherence to operational protocols. | | 76 /107(71.0) |
| C. Patient cooperation and understanding. | | 88 /107(82.2) |
| D. Comprehensive IT systems. | | 79 /107(73.8) |
| E. Nurse motivation. | | 61 /107(57.0) |
| **16. In perspective of patients’ safety , what risks do exist in Internet hospital plus home nursing?(MSQ)** | |  |
| A. Delayed emergency response in case of illness deterioration. | | 97 /107(90.7) |
| B. Increased risk of infections due to non-sterile conditions. | | 86 /107(80.4) |
| C. Limited access to emergency medical equipment and drugs. | | 86 /107(80.4) |
| D. Improper disposal of bio-hazardous medical waste. | | 65 /107(60.8) |
| E. Reduced clinical monitoring and follow-up time. | | 76 /107(71.0) |
| **17. What nurses’ safety do you worry during Internet hospital plus home nursing?(MSQ)** | |  |
| A. Violence from patients or family members. | | 93 /107(86.9) |
| B. Verbal threats. | | 86 /107(80.4) |
| C. Patients with psychiatric conditions. | | 88 /107(80.4) |
| D. Traffic accidents. | | 64 /107(59.8) |
| E. Occupational exposures. | | 76 /107(71.0) |
| **18. What policy support is necessary for Internet hospital plus home nursing?(MSQ)** | |  |
| A. Clear pricing standards and insurance reimbursement policies. | | 98 /107(91.6) |
| B. Standardized service protocols and quality control guidelines. | | 87 /107(81.3) |
| C. Certification and regulation of nurse qualifications. | | 84 /107(78.5) |
| D. Nurse rights and safety protect policies. | | 93 /107(86.9) |
| E. Fair compensation policies for nurses. | | 93 /107(86.9) |
| **19.Which Internet hospital plus home nursing service is potential for expansion?(MSQ)** | |  |
| A. Rehabilitation training. | | 84 /107(78.5) |
| B. Psychological counseling. | | 49 /107(45.8) |
| C. Nutritional support. | | 66 /107(61.7) |
| D. Health education. | | 65 /107(60.8) |
| E. Specialized nursing care. | | 88 /107(82.2) |
| **20. What is the main barrier to expand new Internet hospital plus home nursing items?(MSQ)** | |  |
| A. Low nurse motivation. | | 68 /107(63.6) |
| B. Lack of portable equipment. | | 73 /107(68.2) |
| C. Low patient or family member’s perceiving degree. | | 87 /107(81.3) |
| D. Unclear pricing standards. | | 72 /107(67.3) |
| E. Insufficient insurance coverage. | | 72 /107(67.3) |
| **21. What additional resources can improve Internet hospital plus home nursing?(MSQ)** | |  |
| A. Ambulances with emergency equipment. | | 77 /107(72.0) |
| B. Comprehensive Internet hospital plus home nursing IT system. | | 90 /107(84.1) |
| C. High-definition audio or video recording devices. | | 74 /107(69.2) |
| D. Long-range communicating devices. | | 75 /107(70.1) |
| E. Precision GPS tracking systems. | | 72 /107(67.3) |

1. **Do you have additional suggestions to improve Internet hospital plus home nursing?(OEQ)**

**Note**

1.The content of the questionnaire is comprehensive and detailed. There is no additional suggestion to the 22th question provided by nurses.

2. **SCQ**: Single-choice question(select one answer only); **MSQ**: Multiple-select questions(select all that reply); **OEQ**: Open-ended question(provide written response).
