# Supplementary material for: An Internet Hospital Plus Home Nursing Model for Chronic Disease Patients: Mixed-Methods Study in Tianjin, China
Source: JMIR Nurs. 2025 Nov 5;8:e76761. doi: 10.2196/76761 (PMC12588391; doi:10.2196/76761)
Supplement: Multimedia Appendix 2 [file nursing-v8-e76761-s002.docx]

**Attachment 2**

1. **Comparison of costs between Internet hospital plus home nursing and transferring patients to hospital based on GIS (geographic information system)-based simulation technology**

We utilized the home nursing patient database of TJMUGH to select 498 patients with mobility difficulties (such as fractures, paralysis), and assessed the economic efficiency of home nursing using GIS-based simulation technology.

A cost calculation model was developed based on the officially published pricing standards from the Tianjin Municipal Health Commission and Price Bureau. To address service gaps in its internet hospital and meet the demand for home nursing among patients, TJMUGH launched home nursing through its internet hospital platform in November 2020, established Internet hospital plus home nursing (Internet Hospital Home Nursing) service model. Internet hospital plus home nursing integrated service demands from both online and offline healthcare systems. It adopts a hybrid service framework where patients submit online applications via the platform, followed by nurses delivering offline home nursing services,thereby providing diversified care services tailored to individual needs.

Internet hospital plus home nursing categorizes services into three types: Regular Services, Specialized Services, and Long-Distance Services. Regular Services refer to a single trip serving multiple patients. Specialized Services involve a dedicated trip exclusively for one patient. Long-Distance Services are defined as single-patient trips with distances greater than 10 km but less than 100 km. The basic fees for these services are detailed in S1. Service item fees align with the pricing standards of physical hospital and are charged separately.

**S1.** Basic fees for three types of Internet hospital plus home nursing service (Unit: Yuan)

| Service type | Distance≤3km | 3<Distance≤5 km | 5<Distance≤10 km | 10<Distance≤30 km | 30<Distance≤100km |
| --- | --- | --- | --- | --- | --- |
| Regular services | 80 | 90 | 100 | - | - |
| Specialized services | 140 | 160 | 180 | - | - |
| Long distance services | - | - | - | 300 | 450 |

Note: Regular and specialized service are limited to service distances of 10 km or less, while only long distance service is available for distances greater than 10 km.

The fees for simulated transfer services are categorized by service zones and adhere to the standards of either the non-emergency hotline 96120 or the emergency hotline 120, as detailed in S2. The non-emergency hotline 96120 serves as the exclusive transfer service hotline in Tianjin Municipality. Hotline 96120 and 120 operate in parallel, with transfer service requests independently handled through dedicated call channels, thereby reducing congestion on the emergency hotline 120. Currently, 96120 transfer service is operational in the following districts of Tianjin: Heping, Hedong, Hebei, Hexi, Nankai, Hongqiao, Dongli, Jinnan, and Xiqing. We utilize the emergency hotline 120 for simulated transfer services in other districts

**S2.** Basic fees of patient transfer services via the emergency hotline 120 and non-emergency hotline 96120 (Unit: Yuan).

| Service type | Distance^a^≦3 km | Surcharge/distance^a^ | Floor^b^≦1 | Surcharge/floor^b^ |
| --- | --- | --- | --- | --- |
| Emergency hotline 120 | 50 | 10 | 20 | 10 |
| Non-emergency hotline 96120 | 120 | 10 | 60 | 30 |

^a^ Transportation Fees for Patient Transfer via Emergency Hotline 120 and Non-emergency Hotline 96120.

^b^ Stretcher Service Fees for Patient Transfer via Emergency Hotline 120 and Non-emergency Hotline 96120.

In the field of geographic information simulation modeling, we utilized Python programming to invoke the geocoding/reverse geocoding API of Gaode Map.This enabled the conversion of patient address information WGS84 coordinate system data (with a precision of 1×10⁻⁶degrees), ultimately completing the geospatial calibration for 498 patient cases. The route planning employed a multi-strategy hybrid algorithm, which synchronously acquired three types of path schemes (speed-prioritized, cost-prioritized, and distance-prioritized) through the Amap (Gaode Map) route planning API. The experimental setup incorporated dual time-period simulation parameters for morning peak hours (07:00-12:00) and midday off-peak periods (13:00-18:00), with the shortest feasible paths calculated based on the actual road network topology. The floor parameters were adjusted according to GB50096-2011 (Residential Design Code), with mandatory elevator configuration for buildings of 7 stories or above, and the floor coefficient simplified to 1.

The cost calculation model formulates a composite cost function:

$C_{total} = C_{base} + C_{mileage} + C_{stretcher}$ (1)

$C_{mileage} = r_{m} \times max\left( 0, d-d_{0} \right)$ (2) $C_{stretcher} =C_{s\_base} + r_{f} \times max\left( 0, n-1 \right)$ (3)

In the formula, the function max(0, x) denotes that it takes the value x when x ≥ 0 and 0 otherwise, embodying the tiered characteristics of the pricing rules.The floor correction term max(0, n−1) originates from the base fee for stretcher services already covering first-floor transportation costs. The mileage calculation is determined by the actual road network distance returned through the Amap (Gaode Map) API, rather than the straight-line (Euclidean) distance.

**S3.** Notation.

| Symbol | Definition | Unit(CNY) |
| --- | --- | --- |
| Ctotal | Total cost | Yuan |
| Cbase | Base fare (fixed fee within 3 km) | Yuan |
| Cmileage | Mileage surcharge | Yuan |
| Cstretcher | Stretcher surcharge | Yuan |
| Cs_base | Stretcher service initiation fee | Yuan |
| rm | Excess mileage unit rate | Yuan/km |
| rf​ | Floor surcharge rate | Yuan/floor |
| d | Actual travel mileage | km |
| d0 | Mileage surcharge threshold | 3km |
| n | Patient's floor number | - |

1. **Sensitivity Analyses Using Different Fee Assumptions**

To assess the robustness of our cost comparison findings, we conducted a one-way sensitivity analysis by varying key cost assumptions. We designed nine hypothetical models (M1-M9) to simulate changes in mileage charges (±20%), stretcher fees (±20%), service distance (±2 km), and patient cost-sharing ratios (30% and 50%). The detailed parameters are presented in S4 and S5.

Furthermore, some patients may opt for family-owned vehicles, taxis, shared transport or ride-hailing services as alternative transportation modes. However, for the 498 patients in our study who exhibited symptoms such as mobility impairment and hemiplegia, such options generally lack professional medical support and are inappropriate for patients requiring specialized supervision and continuity of care. Therefore, they were excluded from our primary cost comparison. Future studies may consider evaluating a broader range of transportation scenarios, especially for lower-risk patient groups.

Table 4 outlines the parameter settings for nine hypothetical models (M1-M9) simulating variations in mileage cost, stretcher surcharge, travel distance, and patient cost-sharing ratio. These assumptions were used to test the robustness of ambulance cost estimations.

**S4.** Different fee assumption.

|  | Service type | Distance≦3 km | Surcharge/distance | Floor≦1 | Surcharge/floor |
| --- | --- | --- | --- | --- | --- |
| M1 Baseline | 120 | 50 | 10 | 20 | 10 |
| M1 Baseline | 96120 | 120 | 10 | 60 | 30 |
| M2 mileage costs +20% | 120 | 60 | 12 | 20 | 10 |
| M2 mileage costs +20% | 96120 | 144 | 12 | 60 | 30 |
| M3 mileage costs -20% | 120 | 40 | 8 | 20 | 10 |
| M3 mileage costs -20% | 96120 | 96 | 8 | 60 | 30 |
| M4 stretcher costs +20% | 120 | 50 | 10 | 24 | 12 |
| M4 stretcher costs +20% | 96120 | 120 | 10 | 72 | 36 |
| M5 stretcher costs -20% | 120 | 50 | 10 | 16 | 8 |
| M5 stretcher costs -20% | 96120 | 120 | 10 | 48 | 24 |
| M6 Distance +2km | 120 | 50 | 10 | 20 | 10 |
| M6 Distance +2km | 96120 | 120 | 10 | 60 | 30 |
| M7 Distance -2km | 120 | 50 | 10 | 20 | 10 |
| M7 Distance -2km | 96120 | 120 | 10 | 60 | 30 |
| M8 Patient cost-sharing ratio 30% | 120 | 50 | 10 | 20 | 10 |
| M8 Patient cost-sharing ratio 30% | 96120 | 120 | 10 | 60 | 30 |
| M9 Patient cost-sharing ratio 50% | 120 | 50 | 10 | 20 | 10 |
| M9 Patient cost-sharing ratio 50% | 96120 | 120 | 10 | 60 | 30 |

S5 presents the simulated total costs of ambulance services and the Internet hospital plus home nursing service under different scenarios. DIFF costs indicate the difference between ambulance and Internet hospital plus home nursing costs. Positive values suggest ambulance services are more expensive, while negative values indicate Internet hospital plus home nursing is more costly.

**S5.** sensitivity analyses using different fee assumption.

| Model | Hypothetical mileage costs (yuan) | Hypothetical stretcher costs (yuan) | Simulated ambulance costs (yuan) | Internet hospital plus home nursing costs (yuan) | DIFF costs (yuan) |
| --- | --- | --- | --- | --- | --- |
| **Regular service (5.88 km, 3 floor)** | | | | | |
| M1 | 148.8 | 120 | 268.8 | 100 | 168.8 |
| M2 | 178.56 | 120 | 298.56 | 100 | 198.56 |
| M3 | 119.04 | 120 | 239.04 | 100 | 139.04 |
| M4 | 148.8 | 144 | 292.8 | 100 | 192.8 |
| M5 | 168.8 | 96 | 264.8 | 100 | 164.8 |
| M6 | 148.8 | 120 | 268.8 | 100 | 168.8 |
| M7 | 128.8 | 120 | 248.8 | 100 | 148.8 |
| M8 | 148.8 | 120 | 80.64 | 100 | -19.36 |
| M9 | 148.8 | 120 | 134.4 | 100 | 34.4 |
| **Specialized service (9.87 km, 2 floor)** | | | | | |
| M1 | 188.7 | 90 | 278.7 | 180 | 98.7 |
| M2 | 226.44 | 90 | 316.44 | 180 | 136.44 |
| M3 | 150.96 | 90 | 240.96 | 180 | 60.96 |
| M4 | 188.7 | 108 | 296.7 | 180 | 116.7 |
| M5 | 188.7 | 72 | 260.7 | 180 | 80.7 |
| M6 | 188.7 | 90 | 278.7 | 180 | 98.7 |
| M7 | 188.7 | 90 | 278.7 | 180 | 98.7 |
| M8 | 188.7 | 90 | 83.61 | 180 | -96.39 |
| M9 | 188.7 | 90 | 139.35 | 180 | -40.65 |
| **Long distance service (35.29 km, 2 floor)** | | | | | |
| M1 | 372.9 | 30 | 402.9 | 300 | 102.9 |
| M2 | 447.48 | 30 | 477.48 | 300 | 177.48 |
| M3 | 298.32 | 30 | 328.32 | 300 | 28.32 |
| M4 | 372.9 | 36 | 408.9 | 300 | 108.9 |
| M5 | 372.9 | 24 | 396.9 | 300 | 96.9 |
| M6 | 392.9 | 30 | 422.9 | 300 | 122.9 |
| M7 | 352.9 | 30 | 382.9 | 300 | 82.9 |
| M8 | 372.9 | 30 | 120.87 | 300 | -179.13 |
| M9 | 372.9 | 30 | 201.45 | 300 | -98.55 |
