# Supplementary material for: An Internet Hospital Plus Home Nursing Model for Chronic Disease Patients: Mixed-Methods Study in Tianjin, China
Source: JMIR Nurs. 2025 Nov 5;8:e76761. doi: 10.2196/76761 (PMC12588391; doi:10.2196/76761)
Supplement: Multimedia Appendix 3 [file nursing-v8-e76761-s003.docx]

**Attachment 3**

**S1.** Transferring patients to hospital costs and Internet hospital plus home nursing basic fees (Unit: Yuan).

| Characteristics | Number of patients, n/N (%) | Transferring patients to hospital, mean(SD) | Internet hospital plus home nursing, mean(SD) | Mann-Whitney U test | *P*value |
| --- | --- | --- | --- | --- | --- |
| **Gender** |  |  |  |  |  |
| Men | 227/498 (45.6) | 273.39 (68.18) | 131.54 (64.65) | 2122.00 | <0.001 |
| Women | 271/498 (54.4) | 276.79 (70.35) | 132.29 (60.66) | 2459.50 | <0.001 |
| **Age (years)** |  |  |  |  |  |
| ≤20 | 0/498 (0) |  |  |  |  |
| 20<a≤40 | 12/498 (2.4) | 290.00 (57.05) | 128.33 (45.89) | 0.00 | <0.001 |
| 40<a≤60 | 33/498 (6.6) | 285.76 (69.15) | 128.18 (69.71) | 48.00 | <0.001 |
| 60<a≤80 | 245/498 (49.2) | 285.80 (72.53) | 140.94 (68.77) | 2681.50 | <0.001 |
| >80 | 208/498 (41.8) | 260.29 (63.55) | 122.16 (52.25) | 1165.00 | <0.001 |
| **Number of visits** |  |  |  |  |  |
| One time | 278/498 (55.8) | 274.57 (67.55) | 128.88 (60.64) | 2524.50 | <0.001 |
| Two times | 88/498 (17.7) | 280.68 (71.74) | 138.41 (73.73) | 413.50 | <0.001 |
| ≥Three times | 132/498 (26.5) | 273.03 (71.69) | 134.09 (57.94) | 617.50 | <0.001 |
| **Service distance** |  |  |  |  |  |
| D≤3 | 86/498 (17.3) | 222.91 (47.08) | 96.51 (27.34) | 3.00 | <0.001 |
| 3<D≤5 | 100/498 (20.1) | 247.30 (48.82) | 110.00 (32.72) | 0.00 | <0.001 |
| 5<D≤10 | 193/498 (38.8) | 270.47 (46.91) | 119.64 (35.124) | 0.00 | <0.001 |
| >10 | 119/498 (23.9) | 344.29 (74.58) | 195.97 (85.30) | 1231.00 | <0.001 |
| **Service type** |  |  |  |  |  |
| Regular services | 299/498 (60.0) | 260.13 (53.24) | 93.28 (7.77) | 0.00 | <0.001 |
| Specialized services | 185/498 (37.2) | 283.41 (63.84) | 173.62 (12.18) | 645.50 | <0.001 |
| Long-distance services | 14/498 (2.8) | 490.00 (72.11) | 407.14 (70.32) | 40.00 | 0.007 |

**S2.** Regression results related to transferring patients to hospital costs, Internet hospital plus home nursing basic fees and difference fees.

| Characteristics | Transferring patients to hospital, B^a^ (95%CI) | *P*value | Internet hospital plus home nursing, B^a^ (95%CI) | *P*value | DIFF^b^, B^a^ (95%CI) | *P*value |
| --- | --- | --- | --- | --- | --- | --- |
| **Gender** |  |  |  |  |  |  |
| Men | Reference | -^c^ | Reference | - | Reference | - |
| Women | -0.079 (-8.29-8.13) | 0.990 | -0.28 (-2.33-1.78) | 0.790 | 0.20 (-8.23-8.62) | 0.960 |
| **Age (years)** |  |  |  |  |  |  |
| ≤20 | - | - | - | - | - | - |
| 20<a≤40 | Reference | - | Reference | - | Reference | - |
| 40<a≤60 | -1.20 (-31.77-29.38) | 0.940 | 0.99 (-6.67-8.65) | 0.800 | -2.19 (-33.56-29.18) | 0.900 |
| 60<a≤80 | -4.62 (-31.45-22.21) | 0.740 | 2.12 (-4.60-8.84) | 0.540 | -6.75 (-34.27-20.78) | 0.630 |
| >80 | -10.05 (-37.15-17.05) | 0.470 | 0.86 (-5.93-7.65) | 0.800 | -10.92 (-38.72-16.89) | 0.440 |
| **Number of visits** |  |  |  |  |  |  |
| One time | Reference | - | Reference | - | Reference | - |
| Two times | 6.58 (-4.66-17.83) | 0.250 | 0.95 (-1.85-8.91) | 0.510 | 5.63 (-5.90-17.17) | 0.340 |
| ≥Three times | 4.00 (-8.39-16.39) | 0.530 | 0.16 (-2.95-3.26) | 0.920 | 3.84 (-8.87-16.55) | 0.550 |
| **Service distance** | 7.87 (5.89-9.85) | <0.001 | 2.61 (2.11-3.10) | <0.001 | 5.265 (3.23-7.30) | <0.001 |
| **Service type** |  |  |  |  |  |  |
| Regular services | Reference | - | Reference | - | Reference | - |
| Specialized services | -14.43 (-24.31--4.55) | 0.004 | 73.66 (71.18-76.13) | <0.001 | -88.09 (-98.22--77.95) | <0.001 |
| Long-distance services | -16.97 (-68.33-34.38) | 0.520 | 245.56 (232.69-258.42) | <0.001 | -262.53 (-315.22--209.84) | <0.001 |

^a^B: Unstandardized regression coefficients.

^b^DIFF: Transferring patients to hospital costs - Internet hospital plus home nursing basic fees.

^c^-: Not applicable.
